# Perioperative management with phosphodiesterase type 5 inhibitor and prostaglandin E1 for moderate portopulmonary hypertension following adult-to-adult living-donor liver transplantation: a case report

**DOI:** 10.1186/s40792-018-0423-6

**Published:** 2018-02-07

**Authors:** Takashi Onoe, Asuka Tanaka, Kohei Ishiyama, Kentaro Ide, Hirotaka Tashiro, Hideki Ohdan

**Affiliations:** 1 Department of Gastroenterological and Transplant Surgery, Applied Life Sciences, Institute of Biomedical and Health Sciences, Hiroshima University, 1-2-3 Kasumi, Minami-ku, Hiroshima, 734-0037 Japan; 2 Institute for Clinical Research, National Hospital Organization Kure Medical Center/Chugoku Cancer Center, Kure, Japan

**Keywords:** Cardiac complication, Living-donor liver transplantation, Phosphodiesterase type 5 inhibitor, Portopulmonary hypertension

## Abstract

**Background:**

Portopulmonary hypertension (PPH) is a relatively rare but well-recognized complication of end-stage liver disease. Moderate or severe PPH (mean pulmonary artery pressure [mPAP] ≥ 35 mmHg) is usually a contraindication for liver transplantation due to high operation-related mortality. Here, we report on a patient with moderate PPH whose condition was successfully managed with a phosphodiesterase type 5 (PDE5) inhibitor (tadalafil) and prostaglandin E1, who experienced rapid improvement of PPH after living-donor liver transplantation (LDLT).

**Case presentation:**

A 63-year-old woman with alcoholic decompensated cirrhosis was referred to our hospital for LDLT. She had mild dyspnea on exertion as well as fatigue. Echocardiography and subsequent cardiac catheterization revealed a high mPAP (35 mmHg), and she was diagnosed with moderate PPH. We commenced treatment with oral tadalafil for the PPH. A second preoperative echocardiography demonstrated improved PPH, and she underwent LDLT. An intravenous infusion of prostaglandin E1 was introduced instead of tadalafil during and after the operation.

The mPAP value showed a rapid decrease in mPAP value to 22 mmHg in 2 days. After discontinuation of the prostaglandin E1, the mPAP value remained 23 mmHg. Postoperative catheterization 2 months after LDLT showed no exacerbation of PPH. She was discharged on foot 70 days after LDLT in good condition and has shown a good clinical condition more than 2 years after LDLT.

**Conclusion:**

LDLT could be a radical treatment for PPH with careful management and adequate patient selection. PDE5 inhibitor and PGE1 is effective and feasible for perioperative management of the patient with moderate portopulmonary hypertension in LDLT.

## Background

Portopulmonary hypertension (PPH) is defined by the coexistence of portal and pulmonary hypertension and is a relatively rare but well-recognized complication of end-stage liver disease. PPH is diagnosed in up to 12.5% of patients for whom orthotopic liver transplantation is planned [1]. PPH can be categorized into mild (mean pulmonary artery pressure [mPAP] of 25–34 mmHg) or moderate to severe (mPAP ≥ 35 mmHg) [1, 2]. Without prior attempts to reduce pulmonary pressure, moderate PPH (mPAP 35–50 mmHg) causes an estimated 50% mortality rate after liver transplantation and severe PPH (mPAP > 50 mmHg) may result in a 100% mortality rate [3, 4]. Based on these results, it has been recommended that liver transplantation should not be considered if a patient’s mPAP value is ≥ 45 mmHg; vasodilation treatment before liver transplantation should be considered if the mPAP value is 35–45 mmHg [5].

Phosphodiesterase type 5 (PDE5) inhibitors have recently attracted attention as selective pulmonary vasodilators. Here, we report a case of a patient with decompensated liver cirrhosis complicated by moderate PPH who underwent adult-to-adult living-donor liver transplantation (LDLT) with successful perioperative management using the PDE5 inhibitor, tadalafil in combination with prostaglandin E1, and showed a rapid improvement of PPH after LDLT.

## Case presentation

**Fig. 1:**
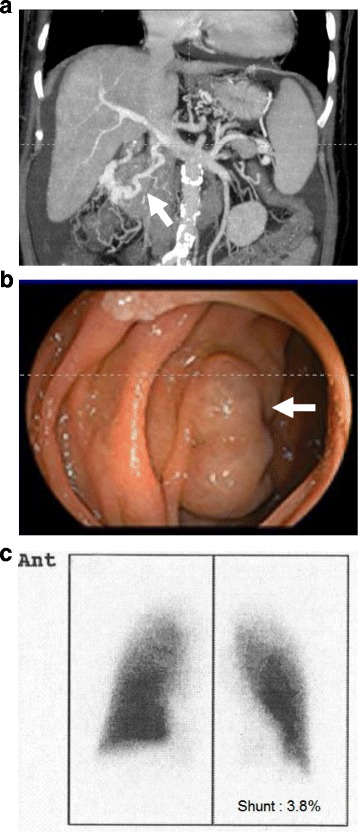
A preoperative coronal section image of CT scan and right spleno-renal shunt (**a** and white arrow, respectively). A preoperative image of upper gastrointestinal endoscopy and varix (**b** and white arrow, respectively). A preoperative radiolabeled technetium macroaggregated-albumin (99mTc-MAA) perfusion scan (**c** anterior image) showing the absence of right to left shunt (3.8%, similar to background)

**Fig. 2:**
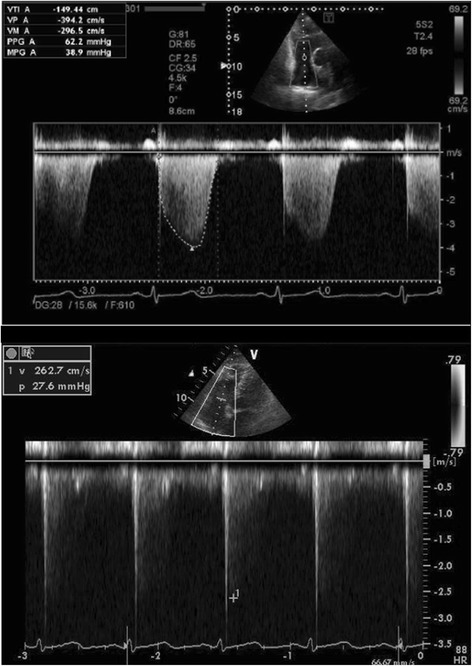
Two-dimensional Doppler echocardiographic images of tricuspid regurgitation 1 month before LDLT (upper) and 2 months after LDLT (bottom)

A 63-year-old woman diagnosed with alcoholic cirrhosis was referred to our hospital for LDLT. The blood tests and physical examination revealed that liver cirrhosis was graded Child-Turcott-Pugh classification C and her calculated model for end-stage liver disease (MELD) score was 17. Preoperative CT scan and endoscopy revealed that she had portosystemic collaterals, such as right spleno-renal shunt and duodenal varix (Fig. 1a, b). She had mild dyspnea on exertion as well as fatigue. Chest radiographs and computed tomography showed mild cardiomegaly (chest-thoracic ratio, 56%); however, no abnormal findings were seen in the lung field. Arterial blood gas (ABG) analysis showed mild hypoxemia at room air (pH, 7.468; partial pressure of carbon dioxide [pCO_2_], 38.3 mmHg; partial pressure of oxygen [pO_2_], 83.0 mmHg), although a radiolabeled technetium macroaggregated-albumin (99mTc-MAA) perfusion scan showed an insignificant right-left shunt of 3.8%, indicating the absence of hepatopulmonary syndrome (HPS; Fig. 1c). Echocardiography revealed tricuspid regurgitation (TR) with estimated mPAP of 54 mmHg, TR-peak pressure gradient value of 62.2 mmHg, and pulmonary vascular resistance (PVR) of 520 dynes s/cm^5^ suggesting pulmonary hypertension (Fig. 2). Heart contractility was normal with normal ejection fraction values of 65%. The patient proceeded to cardiac catheterization, which revealed a high mPAP value (36 mmHg), high pulmonary vascular resistance (476 dynes s/cm^5^), and normal pulmonary capillary wedge pressure (PCWP; 9 mmHg). The patient’s cardiac output (CO) was 4.20 L/min, and cardiac index (CI) was 2.91 L/min/m^2^ (Table 1). Accordingly, she was diagnosed with liver cirrhosis accompanied by moderate-grade PPH. We commenced treatment with oral tadalafil (20 mg/day), an oral once-daily PDE5 inhibitor, for moderate PPH. The patient tolerated the tadalafil therapy well. Twenty-five days after starting tadalafil, a second preoperative echocardiography demonstrated good response to tadalafil and improved PPH with an estimated mPAP of 22 mmHg, TR-peak pressure gradient value of 26.3 mmHg, and PVR of 184 dynes s/cm^5^.

**Table 1:** Perioperative cardiopulmonary hemodynamics

	mPAP (mmHg)	PCWP (mmHg)	CO (L/min)	TR-PPG (mmHg)	PVR (dynes s/cm^5^)	Tadalafil (mg/day)	PGE1 (ng/kg/min)
Pretransplant (42 days before Tx)	54^†^	NA	NA	62.2^†^	520^†^	0	0
Pretransplant (1 month before Tx)	35	9	4.20	NA	476	0	0
Pretransplant (7 days before Tx)	22^†^	NA	NA	26.3^†^	184^†^	20	0
On laparotomy	29	NA	6.9	NA	NA	0	0
On anhepatic phase	23	NA	NA	NA	NA	0	0
Immediately after reperfusion	45	NA	NA	NA	NA	0	0
At the end of surgery	30	NA	7.7	NA	NA	0	7
POD1	27	NA	8.0	NA	NA	0	7
POD2 (before withdrawal of PGE1)	22	NA	6.8	NA	NA	0	7
POD2 (after withdrawal of PGE1)	23	NA	5.8	NA	NA	0	0
POD60	24^†^	NA	NA	28.3^†^	234^†^	0	0
POD62	22	8	4.56	NA	266	0	0

*mPAP* mean pulmonary artery pressure, *PCWP* pulmonary capillary wedge pressure, *CO* cardiac output, *TR-PPG* TR-peak pressure gradient, *PVR* pulmonary vascular resistance, *NA* not applicable, *POD* postoperative day

^†^Values were estimated by echocardiography

Thirty-two days after the initiation of tadalafil, she underwent LDLT with a left lobe graft from her daughter. The graft-versus-recipient weight ratio was 0.76%. Dobutamine hydrochloride (3 μg/kg/min) was administered temporally after graft reperfusion. The pre-explant portal vein pressure was 25 mmHg during surgery and decreased to 17 mmHg after reperfusion. The operation time was 9 h and 56 min, while the total blood loss was 1200 mL. Immunosuppression consisted of methylprednisolone and tacrolimus. An intravenous systemic infusion of prostaglandin E1 (7 ng/kg/min) was started after graft reperfusion and continued intra- and postoperatively instead of tadalafil [6–9]. No hemodynamic events occurred during the operation.

**Fig. 3:**
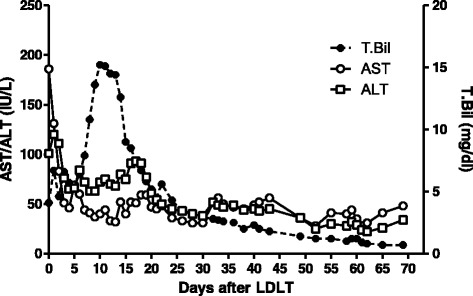
Postoperative course of liver graft function. T.Bil, total bilirubin; AST, aspartate aminotransaminase; ALT, alanine aminotransferase

The mPAP values were continuously monitored using a cardiac catheter intra- and postoperatively, and values rapidly decreased to 22 mmHg 2 days after the LDLT (Table 1). After weaning and discontinuation of the prostaglandin E1, the mPAP value remained 23 mmHg. After mPAP stability was confirmed, the cardiac catheter was removed. Since the mPAP value was effectively reduced, we decided to not subject the patient to a further course of tadalafil. She showed a lot of ascites and hyperbilirubinemia several weeks after LDLT, suggesting mild small-for-size syndrome. This syndrome was gradually resolved by treatment with diuretics and liver graft function gradually improved without rejection (Fig. 3), while a surgical site infection by gram-positive bacillus occurred and she was treated with a course of antibiotics. After LDLT on POD 60, Doppler echocardiography revealed that the TR had disappeared with lowered estimated mPAP, TR-PPG, and PVR value from 54 to 24 mmHg, 62.2 to 27.6 mmHg, and 520 to 234 dynes s/cm^5^, respectively (Fig. 2 and Table 1), suggesting an effective retrieval of right heart function. Right heart catheterization 62 days after the LDLT showed that the mPAP value remained at 22 mmHg with a CO of 4.56 L/min and CI of 3.5 L/min/m^2^, suggesting that the PPH had attenuated (Table 1). ABG analysis showed improved oxygenation at room air (pH 7.393; pCO_2_, 30.8 mmHg; pO_2_, 111.0 mmHg). The patient was discharged 70 days post-LDLT in good condition with no signs of cardiac insufficiency and good liver graft function. She has shown a good clinical course for over 2 years after the LDLT without any evidence of PPH.

An association between portal hypertension and pulmonary hypertension was first reported by Mantz and Craige [10]. PPH is defined as elevated PAP accompanied by hepatic disease and portal hypertension, while the key finding is clearly an elevated calculated PVR [11]. The criteria for the diagnosis of PPH include an elevated resting mPAP value ≥ 25 mmHg, an elevated PVR of ≥ 120 dynes s/cm^5^, normal PCWP (≤ 15 mmHg), and evidence of portal hypertension [12]. However, more recent studies have proposed that the PVR value for the diagnosis of PPH should exceed 240 or 250 dynes s/cm^5^ [13, 14]. The most typical pulmonary symptoms include fatigue, exertional dyspnea, syncope, and chest pain; however, these symptoms are absent in about 60% of patients at the time of diagnosis [15]. Mild hypoxemia at rest due to diffusion limitations and TR may be present on examination. Our patient met the diagnostic criteria of PPH.

PPH is most commonly diagnosed 4–7 years after the diagnosis of portal hypertension [16], and the longer the portal hypertension persists, the greater the risk of PPH exists [16, 17]. The prognosis of patients with PPH is poor, with a mean survival of 15 month [15]. Death occurs due to liver failure, infection, or right heart failure. As mentioned in the “Background” section, liver transplantation in patients with moderate to severe PPH is associated with significantly higher mortality rate [3, 4]. Therefore, several reports have recommended that preoperative estimation of PPH severity by using cardiac catheterization is necessary for the patient selection and preoperative treatment of patients with moderate PPH using vasodilators [1, 5, 18]. Following these recommendations, there have been several reports of successful liver transplantation for patients with moderate to severe PPH using vasodilators in the perioperative period as a bridging therapy, similar to the case reported herein.

Several agents have been used to treat PPH prior to liver transplantation, including epoprostenol [19], bosentan [20], sildenafil [21], iloprost [22], and a combination of these agents [23]. Prostanoids including prostaglandin E1 (alprostadil) and prostacyclin (epoprostenol) are the most widely used and most effective agents for bridging therapy [6–9]. Another agent is the endothelin receptor antagonist, bosentan. Its adverse effect of hepatic dysfunction has limited its use for decompensated liver disease, although it has been seen in only a few patients. Increasing evidence of the efficacy of the PDE5 inhibitor, sildenafil, is emerging, and it has been used for patients with PPH. PDE5 inhibitors boost the effect of endogenous nitrous oxide by inhibiting the breakdown of the messenger substance cyclic guanosine monophosphate, leading to pulmonary vasodilatation and inhibition of vascular smooth muscle proliferation. Selective pulmonary vasodilators rather than systemic vasodilators have demonstrated relatively few adverse effects [24]. Additionally, they are generally well tolerated and have been used successfully for the treatment of pulmonary hypertension [25]. Tadalafil, another PDE5 inhibitor, is a once-daily oral medication that has shown comparably significant pulmonary vasorelaxation in patients with pulmonary hypertension [26]. In our case, we observed successful resolution of PPH using tadalafil without any complications or evidence of toxicity and with good tolerability. Including the present case, only a few reports have described LDLT for adult patients with PPH [27–29] and a few reports have described a combination use of PDE5 inhibitor (sildenafil) and other drugs as bridging therapy in LDLT [30–33].

The patient in this case experienced a rapid improvement of PPH after LDLT. It is debatable whether PPH is reversible after liver transplantation. Most patients with PPH show improved or normalized PAP and can be weaned from vasomodulating medication, which usually takes months [3]. In our case, PPH was rapidly resolved after LDLT. In fact, in our case, we had planned switching back to tadalafil after LDLT; however, the resolution of PPH was more rapid than we had expected. On the other hand, some of patients require long-term treatment due to persistent and progressive pulmonary hypertension after liver transplantation [34]. One speculation for this contradiction is that reversibility might depend on PPH stage. Pathoetiologically, in PPH, vasoactive substances initially cause pulmonary constriction functionally, and subsequently cause endothelial proliferation, and vessel obliteration propagates PPH [35]. Progressive PPH may be organic and irreversible, and its rapid resolution after liver transplantation as seen in our case may be partly attributed by earlier stage PPH. In addition, the responsibility to preoperative treatment with a vasodilator may reflect the stage of PPH. Another speculation is the coexistence of HPS. It has been reported that individual postoperative courses cannot be predicted by the severity of PPH [22]. In some cases, concurrent or sequential onset of PPH with HPS is seen, although its mechanisms are opposite [36]. HPS is a pulmonary vascular dilative process, while PPH is a pulmonary vascular constrictive/obliterative process resulting from portal hypertension [37]. Therefore, PPH might be masked by vasodilation of occult HPS and unmasked by the resolution of HPS after liver transplantation. In our case, occult HPS was absent. Although these are only speculations, these points should be considered and preoperative estimation of HPS and/or responsibility to vasodilator may be important for appropriate selection of liver transplantation candidates.

## Conclusions

Our case demonstrated that moderate PPH can be resolved after adult-to-adult LDLT with appropriate preoperative selection and management with bridging therapy with a combination therapy of PDE5 inhibitor and PGE1. This treatment is effective and feasible for a management of the LDLT recipient with moderate PPH.

## Abbreviations

ABG: Arterial blood gas
CI: Cardiac index
CO: Cardiac output
LDLT: Living-donor liver transplantation
MELD: Model for end-stage liver disease
mPAP: Mean pulmonary artery pressure
pCO_2_: Partial pressure of carbon dioxide
PCWP: Pulmonary capillary wedge pressure
PDE5: Phosphodiesterase type 5
pO_2_: Partial pressure of oxygen
PPH: Portopulmonary hypertension
TR: Tricuspid regurgitation
